# Ineffectual Medical Treatment of Cesarean Scar Ectopic Pregnancy With Systemic Methotrexate: A Report of Two Cases

**DOI:** 10.1177/2324709614528903

**Published:** 2014-03-21

**Authors:** Sefa Kelekçi, Serpil Aydoğmuş, Hüseyin Aydoğmuş, Serenat Eriş, Emine Demirel, Halime Şen Selim

**Affiliations:** 1 Katip Çelebi University Ataturk Training and Research Hospital, Izmir, Turkey

**Keywords:** cesarean scar pregnancy, ectopic, methotrexate, potassium chloride

## Abstract

The implantation of a pregnancy within the scar of a previous cesarean section is known as a “cesarean scar pregnancy.” Its incidence was reported to be 6.1%. However, with the increasing rates of cesarean sections, the incidence is expected to rise. A variety of conservative and surgical treatment modalities have been proposed for the management of cesarean scar pregnancy; however, there are no optimal universal treatment guidelines because of its rarity. Treatment should be tailored to the individual patient. It is obvious that more scar pregnancies will be seen in the future and therefore a set of criteria for the choice of various modes of management should be developed. Here, we present 2 cases of cesarean scar pregnancies treated with a local injection of potassium chloride after the failure of methotrexate administration.

## Introduction

The implantation of a pregnancy within the scar of a previous cesarean section is known as a “cesarean scar pregnancy.” Its incidence was reported by Seow et al^1^ to be 6.1%. However, with the increasing rates of cesarean sections, the incidence is expected to rise. It is believed to develop as a result of the invasion of the myometrium through a microscopic tract between the cesarean section scar and the endometrial canal. The tract is believed to develop from trauma as a result of previous uterine surgeries like dilatation and curettage, myomectomy, metroplasty, and cesarean section.^2^

It can be difficult to differentiate between spontaneous abortion in progress, cervicoisthmic pregnancy, and cesarean scar pregnancy. A high index of suspicion supported by high-resolution ultrasound scanning and an investigation of the patient’s obstetric history is necessary to avoid making the wrong diagnosis. Early diagnosis is essential to avoid catastrophic complications such as uncontrolled hemorrhage and uterine rupture, which may require hysterectomy and result in loss of fertility.

The appropriate treatment of cesarean scar pregnancies is not yet clear. Here, we present 2 cases of cesarean scar pregnancy treated with a local injection of potassium chloride after the failure of methotrexate administration.

## Case 1

**Figure 1.**
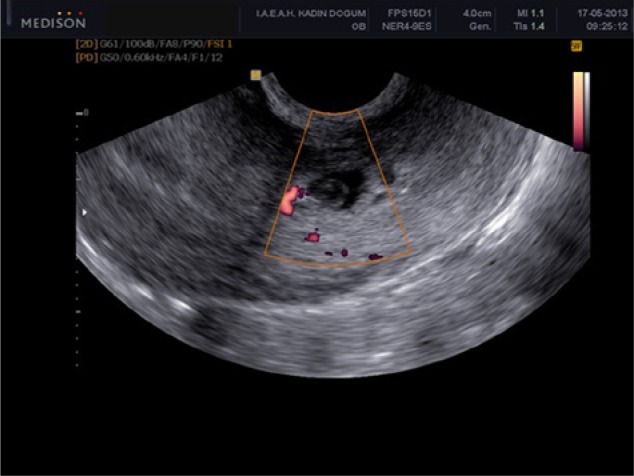
Ultrasound examination of case 1.

**Figure 2.**
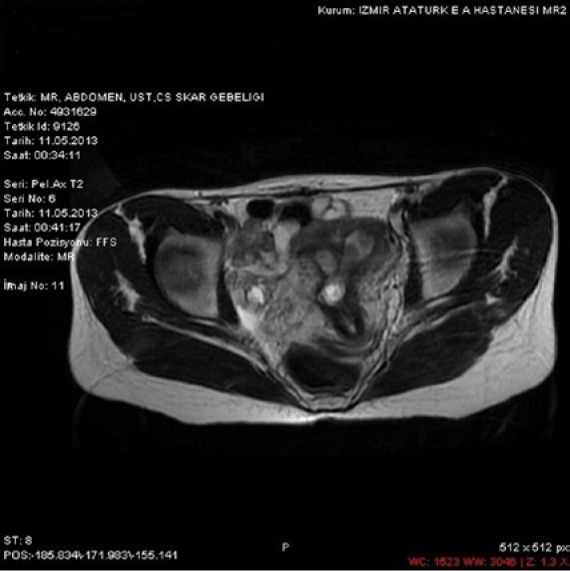
Magnetic resonance imaging examination of case 1.

A 30-year-old, gravida 3 para 2 woman was admitted to our emergency room with the complaint of mild pelvic pain and 5 weeks and 4 days of amenorrhea. The patient’s obstetric history included 2 cesarean sections, the last one being 2 years previously. The first cesarean section was performed for nonprogress in labor and the subsequent one was done electively. At the time of presentation, she was hemodynamically stable. Vaginal examination revealed a closed cervical os, enlarged uterus of 6 weeks’ size, and no adnexal tenderness. Transvaginal ultrasound scan revealed an enlarged uterus, empty uterine cavity and cervical canal; and a gestational sac with a 1.9-mm fetal pole in the lower part of the anterior uterine wall at the site of the cesarean section scar. There was no adnexal mass or fluid in the pouch of Douglas (Figure 1). Serum B-human chorionic gonadotropin (B-HCG) level was 4572 mU/mL. Her magnetic resonance imaging (MRI) examination revealed a bicornuate uterus and a 12-mm gestational sac in the lower part of the anterior uterine wall (Figure 2). The patient was counseled about the diagnosis of a cesarean scar pregnancy. After discussion with the patient, an intramuscular injection of methotrexate (50 mg/m^2^; 70 mg) was given. The dose of methotrexate (70 mg) was repeated on the fourth day of follow-up because the HCG level had risen to 9031.2 mU/mL and the fetal cardiac activity had not ceased. It was an early intervention by us not waiting for the seventh day to repeat the second dose of methotrexate. Three days later, after the second methotrexate dose, the B-HCG level had risen to 9516 mU/mL. Therefore, we performed an ultrasound-guided intrasac injection of 1.5 mL 7.5% potassium chloride with a 20 G spinal needle. On day 5 posttreatment, the serum B-HCG level dropped to 6458 mU/mL. Her pelvic ultrasonogram and serum B-HCG level returned to normal 8 weeks later.

## Case 2

**Figure 3.**
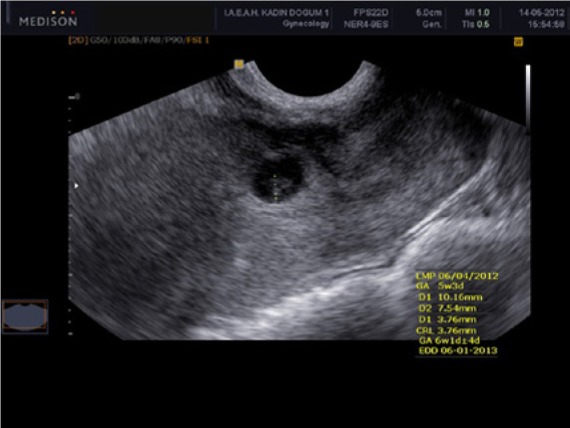
Ultrasound examination of case 2.

A 28-year-old, gravida 4 para 1 woman was admitted to our emergency room with the complaint of vaginal bleeding after 6 weeks and 2 days of amenorrhea. She had had a cesarean section for breech presentation in her first pregnancy 6 years previously. This was followed by 2 missed abortions at 8 weeks and 6 weeks, respectively, for which an evacuation of the uterus was done. Vaginal examination revealed a closed cervical os, 6 weeks–sized uterus, and no adnexal tenderness. On admission, her serum B-HCG level was 4276 mU/mL. A transvaginal ultrasound scan revealed a gestational sac containing a fetus with cardiac activity measuring 3.76 mm (6 weeks 1 days gestation) implanted at the site of her prior cesarean section scar (Figure 3). A diagnosis of a viable cesarean scar pregnancy was made. A single-dose of methotrexate (50 mg/m^2^; 60 mg) was administered intramuscularly. On the fourth day of follow-up, the fetal cardiac activity was still present and serum B-HCG level was 4165 mU/mL. Therefore, we performed ultrasound-guided intrasac injection of 2 mL 7.5% potassium chloride with a 20 G spinal needle and observed an absence of fetal heart activity for 10 minutes during the procedure. On day 2 posttreatment, serum B-HCG level had declined to 3739 mU/mL. Serum B-HCG level declined to within normal range 36 days after initiation of the treatment.

## Discussion

A cesarean section scar pregnancy is a rare condition. The first case was reported in 1978 by Larsen and Solomon.^3^ Since then, cases have been reported with increasing frequency leading to a better understanding of this condition.

Sonography is a reliable first-line diagnostic tool. The following ultrasound criteria have been put forward for the diagnosis of a cesarean scar pregnancy: an empty uterine cavity, without contact with the sac; a clearly visible empty cervical canal, without contact with the sac; the presence of the gestation sac with or without a fetal pole and with or without fetal cardiac activity in the anterior part of the uterine isthmus; and absence of or a defect in the myometrial tissue between the bladder and the sac.^4^

Additional diagnostic information can be obtained by color flow Doppler measurement: High-velocity, prominent, low-impedance blood flow can be detected surrounding an ectopic gestational sac, consistent with normal early pregnancy.^5^

Magnetic resonance imaging has been used as an adjunct to ultrasound scan. MRI is superior in the assessment of the pelvic structures because of the improved differentiation of soft tissue, better spatial resolution, and the possibility of multiplanar imaging. However, a major limitation of MRI is its long acquisition time. Many authors do not routinely recommend MRI for diagnosing cesarean scar pregnancy, and transvaginal ultrasound is still the best diagnostic imaging tool that should be applied in the first step.^6^

A variety of conservative and surgical treatment modalities have been proposed for the management of cesarean scar pregnancy; however, there are no optimal universal treatment guidelines because of its rarity. Treatment should be tailored to the individual patient. The desire for future fertility, size and gestational age of the pregnancy and hemodynamic stability should be considered when determining a treatment plan. Options include wedge resection of the ectopic pregnancy via laparotomy or laparoscopy, hysterescopic excision, local injection of potassium chloride into the sac, and local or systemic methotrexate administration.

Methotrexate is the most common type of medical therapy that is suitable for use in early pregnancy. It may be single or multidose and can be administered systemically or locally. Systemic administration of methotrexate is a standard treatment for tubal ectopic pregnancy, so there should be no reason to doubt its efficacy on cesarean scar pregnancy. But, systemic methotrexate appears to be more effective in women with B-HCG level <5000 mU/mL according to the literature.^7^ Nevertheless systemic methotrexate treatment failed in our cases although serum B-HCG levels were <5000 mU/mL on admission. Continuation of fetal cardiac activity, growth of the sac, and increase in the degree of vascularization with rising B-HCG concentration indicate failure of medical treatment. Intralesional injection and/or other additional interventions are indicated for these patients.^8^ Analysis of published case reports show that medical treatment with methotrexate is successful in 71% to 80% cases with 6% of women requiring hysterectomy.^9^ In one expert review, the complication rate of intramuscular methotrexate without any other treatment in combination was stated as 62.1% so they recommended systemic methotrexate as a single treatment of choice should be avoided. The authors pointed out the slow action of this drug and its questionable ability to stop the cardiac activity and the additional growth of the embriyo/fetus as well as the vascularization of the sac while waiting for the drug effect. In this case, a secondary treatment has to be directed toward a larger gestation with a richer vascularization; thus the risk of complications may increase.^10^ Timor-Tritsch et al^11^ recommended the combination of systemic and intragestational sac administration of methorexate in their case series. We chose the medical treatment with local injection of potassium chloride after methotrexate administration failure rather than surgical treatment. Jurkovic et al^9^ recommended the combination therapy of methotrexate treatment with local injection of potassium chloride in cases with detectable embryonic cardiac activity. Surgical interventions include resection of the ectopic pregnancy or hysterectomy. Advantages of surgical resection are that it provides an opportunity to both remove the pregnancy and repair the defect and avoids the risk of hemorrhage from rupture if medical therapy fails. Expectant management is not a good option because of the risk of rupture and maternal death.^12^ Curettage was considered unsuitable as the first-line treatment option because of the risk of perforation and catastrophic hemorrhage. The reason is probably the fact that unlike the multilayered myometrium in the uterine body, which are able to contain bleeding at the placental site after its separation, the vessels exposed by curettage bleed because the scar tissue is unable to contract and contain the profuse bleeding.^10-13^ Uterine artery embolization has been used to reduce the risk of subsequent hemorrhage in patients who are to undergo conservative surgery or methotrexate injection.^14^

Whether any specific surgical technique such as single or double-layer closure of the incision can minimize or avoid a cesarean scar pregnancy is still an open question. There are numerous articles devoted to the different incision closure techniques. None of them, however, is designed to address whether the type of surgical technique can present cesarean scar pregnancy.^10^

Even if complete resolution of the cesarean scar pregnancy occurs, the patient remains at risk of serious complications such as uterine rupture, placenta accreata, and severe hemorrhage in a subsequent pregnancy. It is advisable that a subsequent pregnancy should be avoided for more than 3 months and preferably a year.

In conclusion, because of the relative rarity of scar pregnancy, it is still unclear which treatment is optimal. It is obvious that more scar pregnancies will be seen in the future and therefore a set of criteria for the choice of various modes of management should be developed.
